# Etiological Trends and Patterns of Antimicrobial Resistance in Respiratory Infections

**DOI:** 10.2174/1874285801812010034

**Published:** 2018-03-30

**Authors:** Salma M. Al-Zain Ahmed, Sara S. Abdelrahman, Doua M. Saad, Isra S. Osman, Modasir G. Osman, Eltahir A. G. Khalil

**Affiliations:** 1 Institute of Endemic Diseases, University of Khartoum, Khartoum, Sudan; 2 Elzahrawi medical laboratory, Khartoum, Khartoum, Sudan

**Keywords:** Etiological trends, Antimicrobial Resistance, Respiratory Infections, Morbidity, Mortality, Nosocomial infection

## Abstract

**Background:**

Respiratory infections are one of the commonest causes of morbidity and mortality related to infectious diseases worldwide. The emergence of antimicrobial resistance is a major global health problem which is well established in developing countries. Good clinical suspicion and correct laboratory identification of respiratory infection causing organisms followed by the appropriate management are needed to compact both community-acquired and nosocomial infection respiratory infections.

**Objectives:**

A retrospective study was carried out to elucidate the etiology of respiratory infections in Sudan, as well as to guide the physician to the best antimicrobial alternatives used in the treatment of respiratory infection.

**Method:**

Respiratory isolates that have been morphologically identified and biologically characterized were subjected to antibiotic susceptibility testing.

**Results:**

A total of 1481 respiratory specimens were examined, recovering 377 organisms from 350 culture positive samples [225(59.7%) sputum, 94(24.9%) broncho-alveolar lavage (BAL), 58(15.4%) Pleural fluid], the commonest organisms were *Klebsiella ssp*. (25.20%) and *mycobacterium tuberculosis* (25.20%), followed by *Staphylococcus aureus*(19.89%) and *Pseudomonas aeruginosa*(8.49%). High rate of resistance of bacterial isolates was observed to Co-trimoxazole (BA), Ampicillin sulbactam (AS), Cefotaxime (CF) and Tetracycline (TE), being 80%, 72.3%, 68.8% and 66.9% respectively; on the other hand, very low resistance rate was found to Amikacin (AK) and Levofloxacin (LE), being 4.6% and 8.5%, respectively.

**Conclusion:**

Guided prescription of antimicrobial agents must be implemented and controlled to limit further spread of antimicrobial resistance.

## 1 INTRODUCTION

Infection of the respiratory tract is regarded as the most common infection among humans worldwide [1]. Respiratory infections are a persistent health problem and being a common reason for consultation and hospitalization, impose an enormous burden on society. The clinical features of respiratory infection differ according to age, sex and co-morbidities [2]. Respiratory infection constituted for 34.6% of reported deaths in the southeast area and out of the total 3,941,000 deaths worldwide [3].

Respiratory infection is a major health problem representing over 50 million deaths per year attributed to both community-acquired and nosocomial infection [4], furthermore respiratory diseases accounted for 13.3% of Disability–Adjusted Life Years (DALYs) [5]. The etiological agents of respiratory infections vary from area to another as well as their antibiotics susceptibility [6]. Among the common bacterial causes of respiratory infections are *Streptococcus*, *Klebsiella, Pseudomonas, Staphylococcus* and *Haemophilus influenza* [7]. However, the responsible pathogens are not identified in 50% of the patients despite thorough diagnostic tests are carried out [8]. Physicians usually rely on clinical signs and symptoms to diagnose respiratory infections; the microbial etiology is rarely identified [9]. Recommendations of therapy are based on the severity of illness; the probabilities of the pathogens in specific geographical areas, resistance patterns of the most commonly implicated etiological agents and co-morbidities [10].

Tuberculosis is a major health problem, nine million new cases of TB and 1.4 million deaths occurred worldwide in 2011. Resistance of *Mycobacterium tuberculosis* to commonly used antituberclus agents is increasing, this adds to the tuberculosis burden [11]. High incidence ranks Sudan among the high prevalence countries for TB, accounting for 14.6% of the total TB burden [12]

The misuse of antibiotics is considered as a direct cause of antibiotic resistance worldwide. Half of the dispensed antibiotics are not truly needed [13].The increased use of over the counter antibiotics not only produces resistance at the individual level but can also threaten the whole community [14].

Antimicrobials should only be prescribed judiciously as the selective pressure on antimicrobial use inevitably leads to increased resistance of the community [15].

Monitoring of antimicrobial susceptibility profile and regulating dispensing of antimicrobials could be effective tools to prevent the spread of antimicrobial resistance [16-19]. Knowledge of agents causing respiratory infections and their antimicrobial profile in Sudan is meager; such information is an important determinant of patient´s treatment. Hence the present study is concerned about the identification of common pathogens causing respiratory infections in Sudan and their pattern of resistance to antimicrobial agents.

## 2 MATERIALS AND METHODS

### 2.1 Study Population

In the present study, a total number of 1481 respiratory samples were collected. The study was carried out for 6 years duration (2009 – 2015), at Elzahrawi medical laboratory-Khartoum state-Sudan, which is a reference laboratory, receives patients from Khartoum & outside of it.

### 2.2 Specimen Collection

All specimens were collected under possible sterile conditions, sputum samples were collected in labeled sterile containers and taken to the laboratory immediately. Bronchoalveolar lavage was obtained by infusion of 20 ml of normal saline solution to bronchial tree, which was then aspirated using fiberoptic bronchoscope. A local anesthetic (lidocaine) was injected into epidermis and parietal pleura, and then another larger gauge needle was inserted into pleural space to withdraw the pleural fluid.

### 2.3 The Microbiological Evaluation

The respiratory samples were inoculated on Mc Conkey, Chocolate and Blood agar (HIMEDIA©) using sterile wire loop; the inoculums were streaked out on the plates, incubated for 24 hours at 37°C and monitored for colonies formation. All isolated bacteria were examined using morphological and biochemical tests following the standard procedure described by Monica Cheesbrough [20]. Samples that are positive for acid and alcohol fast bacilli were referred for a specialized center for culture and sensitivity.

### 2.4 Antimicrobial Susceptibility Testing

Antimicrobial susceptibility was carried out through disk diffusion method (modified Kirby-Bauer method) [20]; disks were obtained from HIMEDIA and AXIOM companies. Antimicrobial agents tracked include: penicillins (penicillin G, amoxicillin/clavulanate, ampicillin/ sulbactam, piperacillin/tazobactam), cephalosporins (cefalexin, cefuroxime, ceftriaxone, ceftazidime, cefoxitin and cefalothin), monobactams (aztreonam), carbapenems (imipenem and meropenem), aminoglycosides (amikacin, gentamycin, lincomycin and streptomycin), fluoroquinolones (ciprofloxacin, norfloxacin, ofloxacin, gatifloxacin, sparfloxacin, pefloxacin, and levofloxacin), sulfonamides (cotrimoxazole), macrolides (erythromycin, roxithromycin), lincomycins (clindamycin),and glycopeptides (vancomycin).

### 2.5 Analytical Tools

Data were analyzed using the Epi Info 7; figures and tables were drawn using Microsoft Excel and Microsoft word.

## 3 RESULT

**Fig. (1):**
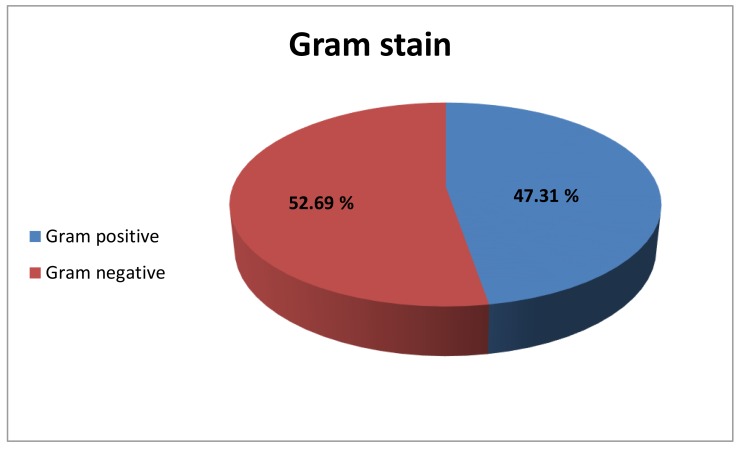
Percentages of gram positive and gram negative bacteria.

Out of 1481 specimens examined, 377organisms recovered from 350 culture positive samples, 225(59.7%), 94(24.9%), 58(15.4%) organisms recovered from sputum, broncho-alveolar lavage (BAL) and Pleural fluid respectively. Male to female ratio was 1.6: 1 (61.8% of cases were males, while 38.2% were females). Using Gram-stain, 52.69% of bacterial isolates were found to be Gram-negative while 47.31% of isolates were Gram-positive (Fig. **1**). The commonest organisms were *Klebsiella* (25.2%) & *Mycobacterium tuberculosis* (25.2%) followed by *Staph aureus*(19.89%) and *Pseudomonas*(8.5%) as shown in Table (**1**).

## 4 DISCUSSION

Out of 1481 samples analyzed, 377 organisms recovered from 350 (23.6%) positive cultures, leaving great number of negative results (1131) (76.4%), which could be attributed to another etiology such as a viral agent or may be due to prior use of the antibiotics.

In the present study, males were 233 (61.8%) and females were 144 (38.2%). The male predominance could be attributed to increasing incidence of smoking and alcohol consumption among males as reported in other studies [21, 22], or probably because females have less access to health care in our country.

Among the bacterial isolates, *Klebsiella pneumoniae* (25.2%) was found to be the predominant organism; the results presented in this paper are in line with those presented in several other papers in India & Nigeria [23-25]. Gram negative bacteria predominated the organisms in this study, similar to the results of H. Farida et al. [26]. Detection of mycobacterium tuberculosis was observed to be high. *Candida* pneumonia was observed to be rare. The presence of *Candida* in sputum or other respiratory tract specimens can be caused by long term antibiotic use, immune suppression secondary to diabetes mellitus or prolonged steroid therapy [27, 28], or often represents contamination [29].

The lowest resistance of bacterial isolates was observed to Amikacin (4.6%) and Levofloxacin (8.5%) Table (**2**), with very low resistance to Amikacin documented in hospital-based study in Egypt [30]. On the other hand, high rate of resistance of bacterial isolates was observed to Co-trimoxazole (BA), Ampicillin sulbactam (AS), Cefotaxime (CF) and Tetracycline (TE): 80%, 72.3%, 68.8% and 66.9% respectively Table (**2**). Our results were in concordance with Victor et al. who reported high resistance of respiratory isolates from Nigeria to AS and BA [31]. The present study showed 90.5%(76/84) and 100%(28/28) resistance of *Klebsiella* and *Pseudomonas aeruginosa* positive isolates to cephalosporins (namely cefotaxime) respectively; this could support intrinsic resistance.

**Fig. (3):**
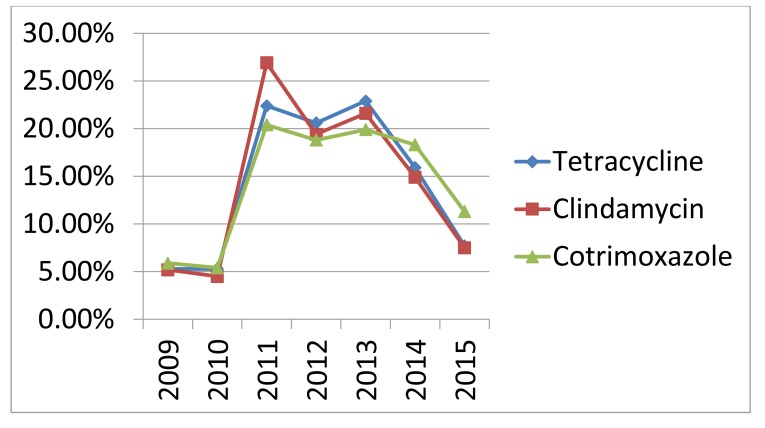
Resistance rate of commonly used antibiotics throughout the years (2009-2015).

**Fig. (4):**
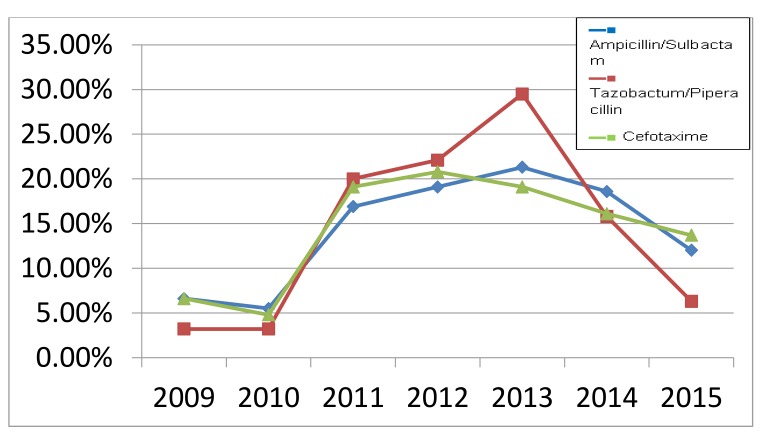
Resistance rate of commonly used antibiotics throughout the years (2009-2015).

**Fig. (5):**
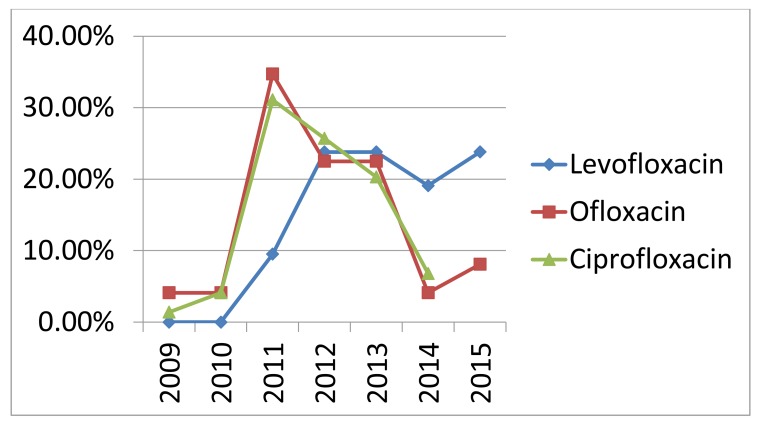
Resistance rate of commonly used antibiotics throughout the years (2009-2015).

Increasing rate of antibiotic resistance throughout the years could be explained by an increase in the consumption of antibiotics. Many studies proved the association between antimicrobial consumption and bacterial resistance [32]. Some antibiotics become sensitive again in later years (Figs. **3** , **4** and **5**), this support the fact that rotation between antibiotic classes reduces the emergence of resistance [33].

## CONCLUSION AND RECOMMENDATIONS

In this study, a high level of resistance to antibiotics was observed. This problem indicates the importance of performing antibiotic susceptibility testing before empirical therapy.

Guided prescription of antimicrobial therapy should be implemented to limit the fast spread of antimicrobial resistance. Public awareness should also be raised to prohibit the widespread antibiotics misuse and to highlight the importance of hygienic practices.

Use of antibiotic cycling policy, as rotation of antibiotic classes, reduces the emergence of resistant organisms.

## Figures and Tables

**Fig. (2):**
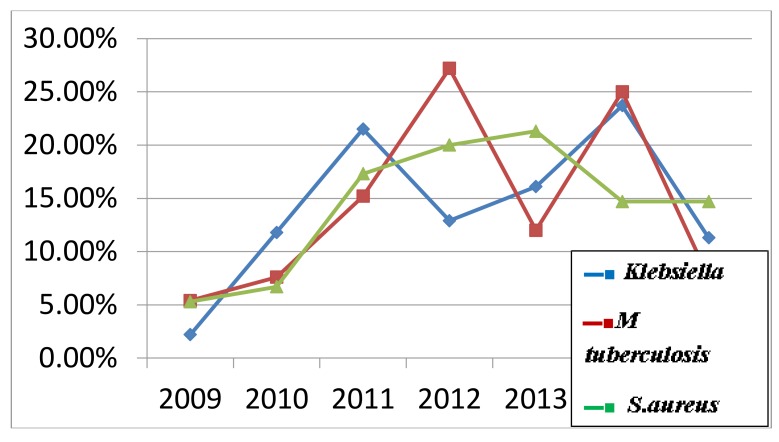
Distribution of organisms throughout the years (2009-2015).

**Table 1:** Frequencies of organisms recovered from respiratory specimens.

**Percentage**	**Frequency**	**Organism**
**25.20%**	**95**	***Klebsiella ssp.***
**25.20%**	**95**	***Mycobacterium tuberculosis***
**19.89%**	**75**	***Staphylococcus aureus***
**8.49%**	**32**	***Pseudomonas***
**4.77%**	**18**	***Streptococcus pneumoniae***
**4.51%**	**17**	***Candida albicans***
**4.24%**	**16**	***Coagulase -ve staphylococci***
**3.45%**	**13**	***Streptococcus pyogenes***
**1.86%**	**7**	***Proteus ssp.***
**1.31%**	**5**	***Aspergillus***
**0.27%**	**1**	***Neisseria gonorrhoeae***
**0.27%**	**1**	***E.coli***
**0.27%**	**1**	***MRSA***
**0.27%**	**1**	**Anaerobic *streptococci***
**100%**	**377**	**Total**

**Table 2:** Percentages of resistant isolates to listed antibiotics.

**Antibiotic class**	**antibiotic**	**No. of G+ve bacteria**	**No. of G-ve bacteria**	**total**	**Percentage of resistant isolates(out of 260)**
**penicillin's**	**AS**	**88**	**100**	**188**	**72.3%**
	**TZP**	**40**	**57**	**97**	**37.3%**
**cephalosporin's**	**CF**	**71**	**88**	**179**	**68.8%**
**quinolons**	**CP**	**44**	**29**	**73**	**28.1%**
	**LE**	**15**	**7**	**22**	**8.5%**
	**OF**	**26**	**21**	**47**	**18.1%**
**aminoglycosides**	**AK**	**9**	**3**	**12**	**4.6%**
	**GM**	**26**	**9**	**35**	**13.5%**
**tetaracyclines**	**TE**	**76**	**98**	**174**	**66.9%**
**chloramphenicol**	**CH**	**29**	**48**	**77**	**29.6%**
**sulfonamides**	**BA**	**85**	**123**	**208**	**80%**
**clindamycin**	**CI**	**53**	**82**	**135**	**51.9%**

Key: AS: Ampicillin sulbactam, TZP: Tazobactum piperacillin, CF: Cefotaxime, CP: Ciprofloxacin, LE: Levofloxacin, OF: Ofloxacin, AK: Amikacin, Gentamycin, TE: Tetracyclin, CH: Chloramphenicol, BA: Cotrimoxazole, CI: Clindamycin. N= 260, this after subtracting *Mycobacterium tuberculosis* (95 isolates), *Candida albicans* (17 isolates) and *Aspergillus* (5 isolates) from the total (377).
